# Multiple micronutrient status and predictors of anemia in young children aged 12-23 months living in New Delhi, India

**DOI:** 10.1371/journal.pone.0209564

**Published:** 2019-02-08

**Authors:** Lisa A. Houghton, Geeta Trilok-Kumar, Deborah McIntosh, Jillian J. Haszard, Michelle J. Harper, Malcolm Reid, Juergen Erhardt, Karl Bailey, Rosalind S. Gibson

**Affiliations:** 1 Department of Human Nutrition, University of Otago, Dunedin, New Zealand; 2 Department of Biochemistry, Institute of Home Economics, University of Delhi, New Delhi, India; 3 Department of Chemistry, University of Otago, Dunedin, New Zealand; 4 VitMin Lab, Willstaett, Germany; University of New Mexico, UNITED STATES

## Abstract

Anemia has been identified as a severe public health concern among young children in India, however, information on the prevalence of anemia attributed to micronutrient deficiencies is lacking. We aimed to assess multiple micronutrient status (iron, zinc, selenium, vitamin A, vitamin D, folate and vitamin B12) in young Indian children and to investigate the role of these seven micronutrients and other non-nutritional factors on hemoglobin concentrations and anemia. One-hundred and twenty children aged 12 to 23 months were included in a cross-sectional nutritional assessment survey, of which 77 children provided a blood sample. Hemoglobin (Hb), serum ferritin, soluble transferrin receptor (sTfR), total body iron, zinc, selenium, retinol binding protein (RBP), folate, vitamin B12 and 25-hydroxyvitamin D (25(OH)D) were measured, and adjusted for inflammation using C-reactive protein (CRP) and α-1-acid glycoprotein (AGP), where appropriate. Predictors for hemoglobin and anemia were identified in multiple regression models. Most of the children were classified as anemic, of which 86 to 93% was associated with iron deficiency depending on the indicator applied. Deficiencies of folate (37%), and notably vitamin D (74%) were also common; fewer children were classified with deficiencies of vitamin B12 (29%), zinc (25%), and vitamin A (17%) and selenium deficiency was nearly absent. Multiple micronutrient deficiencies were common with over half (57%) deficient in three or more micronutrients, and less than 10% of children were classified with adequate status for all the micronutrients measured. Iron status was found to be the only nutritional factor statistically significantly inversely associated with anemia (*P =* 0.003) in multivariate analysis after controlling for sex. A coordinated multi-micronutrient program is urgently needed to combat the co-existing micronutrient deficiencies in these young children to improve micronutrient status and reduce the high burden of childhood anemia.

## Introduction

Anemia has been identified as a severe public health concern (global childhood prevalence ≥ 40%) and in India, recent national anemia prevalence estimates are nearly 60% in children younger than five years [1, 2]. Anemia has significant negative impacts on infant and young child health including impairments in mental, physical and social development, which in later years can result in poor school performance and decreased work productivity [3]. While iron deficiency is thought to be a major cause of anemia, other micronutrients most notably folate, vitamin B12, vitamin A, and more recently, zinc, selenium, and vitamin D, have been associated with childhood anemia [3–6]. Despite several reports of micronutrient deficiencies of vitamin A, zinc, vitamin B12, folate, iron and vitamin D among young Indian children, their relative contribution to the prevalence of anemia remains unknown [7–13].

Accurate assessment of micronutrient status is a critical step in identifying nutritional risk factors for anemia [14]. However, infection and inflammation are known to alter several micronutrient biomarkers, including ferritin, zinc, retinol or retinol binding protein (RBP), and possibly selenium [14, 15]. For example, concentrations of ferritin increase due to subclinical inflammation independent of iron stores whereas retinol, RBP and zinc decline in response to inflammation [14, 16]. Consequently, in young children living in settings in India where there is a high prevalence of infection [17, 18], the true burden of deficiency may be uncertain. Recent efforts led by the Biomarkers Reflecting Inflammation and Nutritional Determinants of Anemia (BRINDA) project have focused on methods for adjusting micronutrient status to account for the effects of inflammation using regression modeling and two acute phase proteins, C-reactive protein (CRP) and α-1-acid glycoprotein (AGP) [14, 16, 19–23]

In this cross-sectional survey of young Indian children aged 12 to 24 months, we aimed to assess the prevalence of anemia and multiple micronutrient deficiencies (iron, zinc, selenium, vitamin A, vitamin D, folate and vitamin B12), adjusted for inflammation where appropriate; and, to investigate the role of these seven micronutrients and other non-nutritional factors on hemoglobin concentrations and anemia.

## Materials and methods

### Study population and design

A convenience sample of 120 children aged 12–23 months living in the slum area Badarpur, New Delhi were surveyed cross-sectionally. Recruitment was conducted from September 2014 to March 2015. Children who were born full term (> 37 weeks) with a birthweight greater than 1500 grams were identified from local Anganwadi records, and the mother or primary caregiver was subsequently contacted by an Anganwadi community worker for participation. Children who had been diagnosed with rickets or who showed evidence of severe disease (active tuberculosis or severe anemia (hemoglobin (Hb) < 70.0 g/L) or acute malnutrition (mid-upper arm circumference < 115mm) were not eligible to participate. A sample of 120 would allow for an estimate of prevalence of deficiency to a precision of at least +/- 10% with 15% missing data. Ethical approval of study was obtained from the University of Otago Human Ethics Committee, New Zealand (H14/094) and Delhi University India (#884). Written informed consent was obtained from the mother or primary caregiver of each child.

### Sociodemographic and anthropometric data collection

Trained Indian research assistants administered a pre-tested questionnaire on socio-demographic characteristics and health status of the child from each participating household. Sociodemographic variables assessed included mother’s age and religion, level of education and employment of parents, wealth index variables, source of household drinking water and type of toilet facility. Health status indicators included infection status (cough, fever and diarrhea in the last two weeks), immunization, and deworming and vitamin A supplementation in the past 6 months. Birth dates of the children were obtained from immunization cards or birth certificates.

Measurements of weight and length for all participants were taken in duplicate using calibrated equipment and standardized techniques [24], with children wearing light clothing and no shoes. Length was measured to the nearest mm using a pediatric length board placed on the floor. Z-scores for length-for-age (LAZ), weight-for-age (WAZ), weight-for-length (WLZ), and Body Mass Index-for-age (BMIZ) were calculated using the World Health Organization (WHO) Child Growth Standards [25] and the computer program WHO Anthro 2011 [26].

### Blood sampling and laboratory analyses

A non-fasting morning peripheral venipuncture blood sample was taken during an organized health camp in March 2015. Blood was drawn into a trace-element (TE)-free evacuated tube (Becton Dickinson, Franklin Lakes, NJ) using International Zinc Nutrition Consultative Group (IZiNCG) procedures [27] and into a second evacuated tube containing EDTA as an anticoagulant (Becton Dickinson, Franklin Lakes, NJ). Time of blood collection and time of the last meal were recorded. All blood samples were refrigerated immediately after collection and the serum from the TE-free tubes separated within two hours using TE-free techniques and protected from ultra-violet light. Aliquots of serum for micronutrient analyses and inflammatory markers were frozen in TE-free polyethylene vials at -80°C prior to shipment on dry ice to the Department of Human Nutrition, University of Otago, New Zealand and to the laboratory of Dr J Erhardt in Germany for analysis.

Hemoglobin was performed by a local laboratory (Dr Lal PathLabs, New Delhi) via automated hematology analyzer (Sysmex XN-1000, USA). Serum ferritin, soluble transferrin receptor (sTfR), RBP, CRP and AGP were analyzed in duplicate by a combined sandwich enzyme linked immunosorbent assay (ELISA) technique [28]. Inter-assay coefficients of variation (CVs) of a pooled plasma sample (n = 40) were 2.3% for ferritin, 3.6% for sTfR, 3.6% for RBP, 5.8% for CRP, and 8.1% for AGP. Total body iron (mg/kg) was calculated as:—[log_10_ (sTfR X 1000 ÷ ferritin) - 2.8229] ÷ 0.1207 [29]. Serum zinc and selenium were analyzed by inductively coupled plasma mass spectrometry (ICP-MS) (Agilent 7500ce ICP-MS, Agilent Technologies, Tokyo, Japan). The CVs for a pooled sample (n = 7) and control (UTAK, Utak Laboratories Inc., Valencia, CA, USA; n = 41) for both zinc and selenium were less than 2%, with the mean results for the control both within 7% of certified values. Serum vitamin B12 was measured using the Cobas 8000 modular analyzer series (Roche Diagnostics, Switzerland) at Southern Community Labs, Dunedin, New Zealand. The lab continually runs the Biorad Immunoassay Plus Control 1 and Control 3 for quality control (QC) of the vitamin B12 immunoassay on the analyzer. Serum folate was analyzed by microbiological assay according to the methods of O’Broin and Kelleher [30] and Molloy and Scott [31] using Lactobacillus rhamnosus (ATCC 7469) and calibration curves produced using 5-methyltetrahydrofolate [(6S)-5-methyl-5,6,7,8-tetrahydropterolyl-L-glutamic acid, sodium salt; Merck Eprova]. A high, medium, and low pooled quality serum control was included on each plate expressed as mean (± 2 standard deviations (SDs)); high 45.5 (11.5) nmol/L; medium 26.3 (5.2) nmol/l; low 15.0 (3.8) nmol/L. Serum total 25-hydroxyvitamin D (25(OH)D_2_ and 25(OH)D_3_) was analyzed by isotope dilution-liquid chromatography-tandem mass spectrometry (API 3200, Applied Biosystems Inc.) based on the method of Maunsell et al [32]. To assess accuracy and inter-assay variability, external QC serum material (UTAK Laboratories, USA) containing low and medium levels of both metabolites were analyzed with every run. Measurements fell within the expected reference ranges for both low and medium controls for 25(OH)D_3_ and 25(OH)D_2_. Internal QC pooled serum samples were also analyzed. The inter-assay CV for 25(OH)D_3_ was 0.7% at 52.7 nmol/L, whereas 25(OH)D_2_ was below the limit of quantification.

The following interpretative criteria were used to define the risk of micronutrient deficiencies: anemia, Hb < 11.0 g/dL [33]; serum ferritin < 12 μg/L [34]; sTfR > 8·3 mg/L [28]; total body iron stores < 0 mg/kg [29]; RBP < 0·83 μmol/L [35]; serum zinc < 9·9 μmol/L [36]; serum folate < 6.8 nmol/L [37], vitamin B12 < 221 pmol/L (insufficient) and < 148 pmol/L (moderate deficiency) [38]; 25(OH)D < 50 nmol/L [39]; selenium ≤ 0·82 μmol/L [40]. We calculated the prevalence of iron deficiency anemia (IDA; concomitant iron deficiency plus anemia) and the proportion of anemic children with iron deficiency based on all three iron status indicators.

### Statistical analyses

Descriptive statistics were calculated for sociodemographic data, child health characteristics and micronutrient biomarkers. An asset-based wealth index was calculated using principal component analysis (PCA) based on Demographic and Health Survey Wealth Index guidelines [41] and asset variables recommended for use in the National Family Health Survey (NFHS-3) [42]. This continuous index was then divided into quintiles from the lowest to highest household wealth. Serum ferritin, sTfR, body iron, RBP, zinc and selenium were adjusted for subclinical inflammation using the recently recommended regression modelling approach developed by the BRINDA Project [14]. Adjustments by the BRINDA method were undertaken using a linear regression model with each micronutrient biomarker as the dependent variable (all log-transformed because of positive skew); and CRP and AGP as the independent variables. The slope (regression coefficient) of CRP (β1) and AGP (β2) was then used to adjust for the effect of inflammation as follows: exp[unadjusted ln(biomarkers)– β1 (CRP_observed_−maximum of lowest decile for CRP)– β2 (AGP_observed_−maximum of lowest decile for AGP)]. A reference concentration (maximum of lowest decile) for serum CRP and AGP was used to avoid over-adjusting the micronutrient biomarkers among individuals with low levels of inflammation. All models were checked to ensure all assumptions were met by examining the plot of residuals, homogeneity of variance, and normality. Adjustments for serum zinc for time of blood collection and time since last meal prior to the blood collection were made before adjusting for inflammation based on the method of Arsenault et al [43].

Mean and SD or geometric mean (GM) (95% confidence interval (CI)) were calculated for each biomarker variable, both unadjusted and inflammation-adjusted where appropriate. If data exhibited positive skew, geometric means were presented. Univariate linear regression models with age, sex, maternal education, wealth index, inflammation factors, and biomarkers as predictors were used to examine unadjusted associations with hemoglobin. Multiple linear regression was then used to identify the independent micronutrient predictors of hemoglobin. The explanatory variables included in the model were all micronutrients and covariates that were known or suspected to be biologically important from previous literature or from the univariate models. Model assumptions were checked using residual plots and there was no evidence in the models of collinearity (VIF > 2) among the independent variables. Standardized micronutrient and ln(CRP) values were also used in the models to allow comparison of the strength of association with hemoglobin between predictors. As adjusted sTfR exhibited strong positive-skew it was log transformed before standardization. Only the strongest predictor out of ferritin, body iron, and sTfR was included in the adjusted model determined by the highest standardized coefficient from the univariate models and R^2^. Logistic regression analysis was performed to determine the odds ratio (OR) and 95% CI for the children with anemia. Unadjusted and adjusted models were run as for the hemoglobin analysis, however as the multivariate logistic model was limited by the small number of non-anemic children only the strongest predictor variables with *P*< 0.25 from the univariate analyses were included. Goodness of fit was assessed with a Hosmer-Lemeshow test with groups of 10. Statistical analyses were carried out using Stata 13.1 [44]. A *P*-value of < 0.05 indicated statistical significance.

## Results

Of the 120 children enrolled in the study, 77 provided a blood sample constituting the final sample size for the present study. Given there were more missing data from the blood samples than expected (36% instead of 15%), the power to determine the prevalence of deficiency with biomarkers was reduced to a 95% precision level of 11% (instead of 10%). Socio-demographic characteristics of participants are presented in Table 1. The average age of the children was 17.4 ± 3.6 months and both sexes were equally represented. When comparing those children who provided a blood sample and those who did not, we found no significant difference in child age, mother’s education, marital status and parity; however, mothers who consented to providing a child blood sample were older (26.5 ± 4.2 years versus 25.0 ± 3.2 years, respectively; *P =* 0.049) and a larger percentage were Hindu (93.5% versus 81.4%, respectively; *P =* 0.041). Moreover, a higher proportion of children who gave blood received vitamin A supplements in the previous 6 months (67.5% vs 46.5%, respectively; *P =* 0.024) and had slightly higher height-for-age z-score than those who did not provide a blood sample (-1.53 ± 1.11 vs. -1.97 ± 1.27, respectively; *P =* 0.0497).

**Table 1:** Sociodemographic and household characteristics.

Variables	n	%
Child age (months)	77	17.4 ± 3.6^1^
Mothers age (years)	77	26.5 ± 4.2^1^
Sex (Female)	34	44.2
Birth order		
1^st^	33	43.4
2^nd^	23	30.3
3^rd^	13	17.1
4^th^	7	9.2
Under 5		
1 child	50	64.9
2	25	32.5
3 or more	1	1.3
Religion		
Hindu	72	93.5
Muslim	3	3.9
Sikh	2	2.6
Christian	0	0
Highest level of schooling of mother		
Did not study	15	19.5
Some primary	10	13.0
Some secondary	40	52.0
University	12	15.6
Highest level of schooling of father		
Did not study	3	3.9
Some primary	7	9.1
Some secondary	49	63.6
University	18	23.4
Main source of drinking water		
Piped into dwelling	69	89.6
Piped to public tap/standpipe	4	5.2
Purchased water from market	1	1.3
Tubewell or borehole	1	1.3
Piped into yardplot	2	2.6
Type of toilet facility		
Flush to septic tank	72	94.7
Flush to piped sewer system	2	2.6
Other	2	2.6
Share toilet with other households	52	67.5

^1^mean±SD

Overall, the majority of children were fully immunized (> 90%) and two thirds had received vitamin A supplements in the previous 6 months (Table 2). In contrast, fewer children received deworming treatment. Morbidity symptoms were reported in approximately one-third of children, with cough or fever in the past two weeks being the predominant symptoms. The prevalence of elevated AGP (39%) was higher than that of CRP (16%); however, more than half (61%) the children had concentrations of AGP and CRP below the respective cut-offs. Growth failure was evident in the children with a high prevalence of stunting (33%) and underweight (22%), whereas the prevalence of both wasting and thinness (BMIZ < -2) was low (< 10%); no child had a BMIZ > 2 indicative of childhood obesity (Table 2).

**Table 2:** Health and anthropometric characteristics of young children.

Variables	n	%^1^
Illness in past 2 weeks		
Diarrhea	18	23.4
Cough	26	33.8
Fever	24	31.2
Vomiting	5	6.5
Vaccinations		
Tuberculosis (BCG)	75	97.4
Polio	74	96.1
DPT	75	97.4
Measles	72	93.5
Hepatitis B	71	92.2
Deworming treatment in past 6 months	14	18.2
Vitamin A syrup in past 6 months	52	67.5
Plasma CRP, geometric mean (95% CI)	75	0.71 (0.51, 0.99)
>5 mg/l	12	16.0
Plasma AGP, geometric mean (95% CI)	75	0.88 (0.78, 1.00)
>1 g/L	29	38.7
Stage of inflammation^2^		
Apparently healthy	46	61.3
Incubation	0	0
Early convalescence	12	16.0
Late convalescence	17	22.7
LAZ, mean ± SD	76	-1.53 ± 1.11
Stunting (LAZ < -2 (95% CI))	25	32.9 (22.5, 44.6)
WAZ, mean ± SD	76	-1.25 ± 1.14
Underweight (WAZ <-2 (95% CI))	17	22.4 (13.6, 33.4)
WLZ, mean ± SD	76	-0.71 ± 1.06
Wasting (WLZ < -2 (95% CI))	7	9.2 (3.8, 18.1)
BMIZ, mean ± SD	76	-0.46 ± 1.02
Thinness (BMIZ < -2 (95% CI))	4	5.3 (1.5, 12.9)
Overweight (BMIZ >2 (95% CI))	0	0 (0, 4.7)

Abbreviations: DPT, diphtheria, pertussis, and tetanus; CRP, C-reactive protein; AGP, α-1-acid glycoprotein; LAZ length-for-age Z score; WAZ, weight-forage Z score; WLZ, weight-for-length Z score, BMIZ, body mass index-for-age Z score

^1^Unless otherwise noted

^2^Stage of inflammation: Healthy, CRP ≤ 5 mg/L and AGP ≤ 1 g/L; Incubation, CRP > 5 mg/L and AGP ≤ 1 g/L; early convalescence, CRP > 5 mg/L and AGP > 1 g/L; late convalescence, CRP ≤ 5 mg/L and AGP > 1 g/L

Micronutrient status indicators and the prevalence of deficiency are shown in Table 3. The geometric mean values for serum ferritin and sTfR were lower when taking into account inflammation (*P*< 0.001), whereas those for body iron, RBP and selenium were higher (*P*< 0.001). The concentration of serum zinc after adjustment for time of last meal and the blood drawing was significantly lower compared to the unadjusted mean zinc concentration (*P*< 0.001); subsequent adjustment to account for inflammation significantly elevated serum zinc concentrations (*P*< 0.001) and markedly lowered the prevalence of deficiency. Most of the children were classified as anemic, of which 86 to 93% was associated with iron deficiency depending on the indicator applied. Deficiencies of folate (37%), and notably vitamin D (74%) were also common; fewer children were classified with deficiencies of vitamin B12 (29%), zinc (25%), and vitamin A (17%) after inflammation-adjustment, where appropriate, and selenium deficiency was nearly absent. Multiple micronutrient deficiencies were common with over half (57%) deficient in three or more micronutrients, and more than one-third (39%) deficient in one or two micronutrients. Less than 10% of children were classified with adequate status for all the micronutrients measured.

**Table 3:** Micronutrient and anemia status by different iron indicators.

	n	Geometric Mean (95% CI)	Cut-off	% deficient (95% CI)	% IDA	Anemia associated with ID^1^
Hb, g/dL	77	9.7 (9.4, 10.0)	< 11.0	79.2 (68.5, 87.6)	-	-
Ferritin, ug/L	75		< 12			
Unadjusted		7.8 (6.6, 9.3)		73.3 (61.9, 82.9)	68.0 (56.2, 78.3)	85.9%
Regression correction		7.4 (6.2, 8.8)		73.3 (61.9, 82.9)	68.0 (56.2, 78.3)	85.9%
sTfR, mg/L	75		> 8.3			
Unadjusted		13.9 (12.3, 15.70		82.7 (72.2, 90.4)	73.3 (61.9, 82.9)	92.6%
Regression-correction		12.5 (11.0, 14.1)		73.3 (61.9, 82.9)	68.0 (56.2, 78.3)	85.9%
Body iron^2^, mg/kg	75		< 0			
Unadjusted		-3.5 (-4.5, -2.6)		78.7 (67.7, 87.3)	72.0 (60.4, 81.8)	90.9%
Regression-correction		-3.3 (-4.3, -2.4)		77.3 (66.2, 86.2)	72.0 (60.4, 81.8)	90.9%
RBP, μmol/L	75		< 0.83			
Unadjusted		0.97 (0.91, 1.03)		22.7 (13.8, 33.8)	-	-
Regression-correction		1.07 (1.01, 1.14)		17.3 (9.6, 27.8)	-	-
Selenium, μmol/L			≤ 0.82			
Unadjusted	77	1.09 (1.06, 1.12)		1.3 (0.0, 7.0)	-	-
Regression-correction	75	2.94 (2.84, 3.04)		0.0 (0.0, 4.8)	-	-
Zinc, μmol/L			< 9.9			
Unadjusted	77	11.1 (10.7, 11.4)		23.4 (14.5, 34.4)	-	-
Time adjustment	74	10.4 (10.0, 10.8)		37.8 (26.8, 49.9)	-	-
Regression-correction	72	10.8 (10.4, 11.1)		25.0 (15.5, 36.6)	-	-
Vitamin B12, pmol/L	77	328 (287, 375)	< 221	29.8 (20.0, 41.4)	-	-
			< 148	9.1 (3.7, 17.8)		
25(OH)D, nmol/L	77	32.3 (28.3, 37.0)	< 50	74.0 (62.8, 83.4)	-	-
Serum folate, nmol/L	75	8.1 (7.1, 9.1)	< 6.8	37.3 (26.4, 49.3)	-	-
Mean cell volume, fL	77	68.3 (66.5, 70.1)	< 75	80.5 (69.9, 88.7)	-	-

Abbreviations: Hb, haemoglobin; sTfR, soluble transferrin receptor; RBP, retinol binding protein; 25(OH)D, 25-hydroxyvitamin D; IDA iron deficiency anemia; ID iron deficiency

^1^Percentage calculated by dividing prevalence of IDA for the iron indicator by the overall prevalence of anemia (79.2% in 77 children)

^2^Arithmetic mean (95% CI)

### Determinants of hemoglobin and anemia

In the univariate regression model, none of the sociodemographic characteristics (child age, sex, maternal education and wealth index) were significantly associated with hemoglobin. Ferritin, body iron, RBP and 25(OH)D had statistically significant positive associations with hemoglobin, whereas for sTfR, the association was significantly negative (Table 4). However, the multivariate regression model shows that only elevated concentrations of sTfR and CRP were independently associated with lower hemoglobin concentrations. sTfR was chosen as the iron indicator in the final model on the basis of a higher R^2^ of 67.3% compared with body iron (R^2^ = 60.0%) and ferritin (R^2^ = 30.0%); albeit both ferritin and body iron remained significant in the final multivariate model when tested (both *P*< 0.001, data not shown). Notably, vitamin D, while strongly associated in the univariate model with hemoglobin, was rendered non-significant in the fully adjusted model.

**Table 4:** Regression analysis of sociodemographic and micronutrient predictors of hemoglobin concentration (g/L) (n = 77).

	Unadjusted			Adjusted^1^ (n = 72)		
	B coefficient (95% CI)	Standardized coefficient^2^ (95% CI)	*P*-value	B coefficient (95% CI)	Standardized coefficient^2^ (95% CI)	*P*-value
Sex (male)	-0.25 (-0.88, 0.38)	-	0.439			
Age (months)	-0.05 (-0.14, .04)	-	0.256			
Maternal education (trend across categories)	-0.16 (-0.42, 0.23)	-	0.555			
Wealth index (trend across quintiles)	-0.02 (-0.24, 0.20)	-	0.857			
Adjusted ferritin, μg/L	0.09 (0.06, 0.12)	0.75 (0.49, 1.02)	<0.001			
Adjusted body iron, mg/kg	0.25 (0.20, 0.30)	1.07 (0.86, 1.27)	<0.001			
Adjusted sTfR, mg/L	-0.13 (-0.15, -0.11)	-1.13 (-1.31, 0.94)^3^	<0.001	-0.13 (-0.15, -0.10)	-1.09 (-1.31, -0.87)^3^	<0.001
Adjusted RBP, μmol/L	1.50 (0.44, 2.55)	0.43 (0.13, 0.74)	0.006	0.50 (-0.21, 1.21)	0.19 (-0.02, 0.39)	0.162
Adjusted zinc, μmol/L	0.05 (-0.13, 0.24)	0.09 (-0.24, 0.42)	0.577	0.01 (-0.10, 0.12)	-0.04 (-0.23, 0.16)	0.844
Adjusted selenium, μmol/L	0.08 (-0.66, 0.81)	0.03 (-0.29, 0.35)	0.839	0.27 (-0.27, 0.82)	0.13 (-0.11, 0.37)	0.319
Folate, nmol/L	0.04 (-0.01, 0.10)	0.23 (-0.08, 0.55)	0.145	0.03 (-0.01, 0.06)	0.09 (-0.13, 0.31)	0.180
Vitamin B12, pmol/L	-0.001 (-0.002, 0.001)	-0.14 (-0.45, 0.18)	0.383	0.000 (-0.001, 0.001)	-0.11 (-0.34, 0.12)	0.660
25(OH)D, nmol/L	0.03 (0.01, 0.04)	0.53 (0.23, 0.82)	0.001	0.00 (-0.01, 0.01)	0.01 (-0.21, 0.22)	0.909
Ln-CRP, mg/L	-0.14 (-0.36, 0.08)	-0.20 (-0.52, 0.12)	0.212	-0.18 (-0.31, -0.06)	-0.22 (-0.41, -0.04)	0.005

Abbreviations: sTfR, soluble transferrin receptor; RBP, retinol binding protein; 25(OH)D, 25-hydroxyvitamin D; CRP, C-reactive protein

^1^Final model using iron indicator soluble transferrin receptor (R^2^ = 73.7%); all variables included in the adjusted analysis are those with estimates presented

^2^Standardized coefficients were found by standardizing the predictor variables and interpreted as: i.e., 1 SD higher adjusted RBP was associated with 0.43 g/L higher hemoglobin concentration in an unadjusted model

^3^Standardized adjusted sTfR values were determined using the log-transformed value of adjusted sTfR due to large positive-skew

Further exploration of the relation between vitamin D and hemoglobin before and after adjustment was carried out. It was found that 25(OH)D and sTfR were negatively related (B = -0.18, 95% CI: -0.27, -0.10, *P*< 0.001), illustrating that sTfR is an important confounder of the association between sTfR and hemoglobin.

Of the factors examined in logistic regression analysis with anemia as the outcome variable, iron status was found to be the only dietary factor statistically significantly inversely associated with anemia (*P =* 0.003) in multivariate analysis after controlling for sex (Table 5). Mean sTfR concentration for anemic and non-anemic children was 16.1 ± 8.7 and 7.8 ± 2.3, respectively (*P*< 0.001). Selenium was also found to be inversely associated with anemia albeit non-significant (adjusted analysis: *P =* 0.062). Folate, vitamin B12, vitamin D, and zinc were not included in the final model as they were the least significant in the univariate analysis and sample size was too small to accommodate these variables.

**Table 5:** Logistic regression analysis of sociodemographic and micronutrient predictors of anemia (n = 77).

	Unadjusted			Adjusted^1^ (n = 75)		
	OR (95% CI)	Standardized^2^ OR (95% CI)	*P*-value	OR (95% CI)	Standardized^1^ OR (95% CI)	*P*-value
Sex (male)	2.57 (0.83, 7.99)		0.103	3.20 (0.63, 16.22)	3.09 (0.61, 15.6)	0.160
Adjusted ferritin, μg/L	0.87 (0.81, 0.94)	0.32 (0.17, 0.61)	<0.001			
Adjusted body iron, mg/kg	0.64 (0.50, 0.81)	0.15 (0.05, 0.40)	<0.001			
Adjusted sTfR, mg/L	1.54 (1.17, 2.02)	8.14 (2.5, 26.8)^3^	0.002	1.63 (1.19, 2.24)	10.8 (2.60, 44.8)^2^	0.003
Adjusted RBP, μmol/L	0.18 (0.03, 1.26)	0.61 (0.35, 1.07)	0.084	0.15 (0.01, 3.12)	0.56 (0.24, 1.35)	0.222
Adjusted zinc, μmol/L	0.84 (0.61, 1.15)	0.73 (0.42, 1.27)	0.270			
Adjusted selenium, μmol/L	0.35 (0.10, 1.27)	0.63 (0.36, 1.11)	0.110	0.17 (0.02, 1.10)	0.46 (0.20, 1.05)	0.062
Folate, nmol/L	0.97 (0.89, 1.07)	0.85 (0.50, 1.44)	0.549			
Vitamin B12, pmol/L	1.00 (1.00, 1.00)	0.83 (0.49, 1.41)	0.487			
25(OH)D, nmol/L	0.99 (0.96, 1.01)	0.76 (0.45, 1.31)	0.324			
Ln-CRP, mg/dL	1.55 (0.94, 2.54)	1.89 (0.92, 3.89)	0.058	2.19 (0.96, 4.99)	3.13 (0.96, 10.2)	0.062

Abbreviations: OR, odds ratio; sTfR, soluble transferrin receptor; RBP, retinol binding protein; 25(OH)D, 25-hydroxyvitamin D; CRP, C-reactive protein

^1^All variables included in the adjusted analysis are those with estimates presented

^2^ Standardized ORs were found by standardizing the predictor variables and interpreted as: i.e., 1SD higher adjusted RBP was associated with 0.61 g/L higher hemoglobin concentration in an unadjusted model

^3^ Standardized adjusted sTfR values were determined using the log-transformed value of adjusted sTfR due to large positive-skew

## Discussion

In our sample of young New Delhi children aged 12–24 months, there is striking evidence of undernutrition, morbidity, anemia and multiple micronutrient deficiencies. Less than 10% of the young children had no deficiencies based on the seven micronutrients examined, with 77% at risk of ≥ two co-existing micronutrient deficiencies. Anemia in this sample of young children is also of severe public health concern [2], and approximately 20% higher than the recent national estimate for Indian children under 5 years [1]. Most of the anemia in this sample was explained by iron deficiency, and complex interrelationships between iron and vitamin D on hemoglobin concentrations were also found, highlighting the difficulty of identifying determinants of anemia in a setting where multiple micronutrient deficiencies co-exist.

It should also be noted that adjustment of iron, zinc, RBP, and selenium for elevated CRP and AGP using the regression-correction approach altered concentration levels. This highlights the importance of measuring inflammatory markers when assessing micronutrient status in settings where inflammation and infection is prevalent. Of particular note, inflammation adjustment of both sTfR and zinc led to the greatest decrease in the prevalence of deficiency. Previously, sTfR and zinc were not considered to be influenced by inflammation on the basis that they were not acute phase proteins like ferritin. However, several reports have described inflammatory-induced changes in sTfR [22, 45–48] and zinc [49–52] thereby suggesting an alternative influence of the inflammatory response [53–55]

The extent of both iron deficiency and IDA varied depending on the iron indicator used as did the proportion of anemia associated with iron deficiency. The prevalence of iron deficiency was greatest as measured by total body iron compared with sTfR and ferritin. Total body iron reflects the size of the iron deficit and is the only method that is derived from actual experimental observations, albeit in adults only [29]. Comparisons between studies are limited by the use of different indicators, cut-offs and lack of adjustment for inflammation; however, the very high prevalence of iron deficiency identified in our study was consistent with that observed for rural children of a similar age in Punjab, India (71.8% of children as defined by unadjusted low ferritin < 10 g/L) [56] and of preschool children living in Karnataka, India (61.9% as defined by low ferritin < 12 ng/mL; < 30 ng/mL if CRP > 5 mg/L) [11]. In contrast, a study of young children aged 6 to 30 months in low- to middle-income neighborhoods in New Delhi reported a much lower prevalence of iron deficiency (31%) as defined by unadjusted elevated sTfR (> 4.7 nmol/L). The authors of this latter study, however, did not measure inflammation and excluded children with reported severe infection [12].

Unlike iron, selenium deficiency was not evident among our study population, probably reflecting the normal or above normal concentrations of plant-available selenium in the soils, and hence plant-based staple foods grown in the Northern districts of India [57, 58]. Interestingly, despite a seemingly adequate status, having an even higher selenium concentration was independently associated with a reduced risk of anemia in our sample of children albeit non-significant. Associations between low serum selenium and anemia have been reported in school children and older adults alike [5, 59]. Selenium is an integral part of the enzyme glutathione peroxidase that has an important antioxidant function including protection of hemoglobin [59]. Another potential mechanism through which selenium could influence anemia is by modulating inflammatory pathways and regulating hepcidin [59].

In the present study, we did not find zinc to be an independent predictor of hemoglobin concentrations. The lower prevalence of zinc deficiency compared to iron (25% vs. > 70%) reported here is somewhat surprising given that iron and zinc have a similar distribution in the food supply and absorption of each are affected by some of the same food components [27]. Nevertheless, stunting, a well-recognized feature of zinc deficiency during childhood, was high (33%) among our sample population and our stunted children did have lower mean serum zinc concentrations than their non-stunted counterparts [mean difference (95% CI): -0.5 (-1.4, 0.4) μmol/L, *P =* 0.258]. While national-level data on zinc status are limited, some but not all studies in India highlight the likelihood of widespread zinc deficiency among preschool children. In five major Indian states, approximately 50% of children aged 6–60 months were shown to be zinc deficient [8] whereas smaller surveys report less than 20% of children aged 6–60 months with zinc deficiency despite a high prevalence of low serum ferritin levels (72%) and raised sTfR (80%) [56, 60].

The prevalence of vitamin A deficiency among the children was low (17%) and no longer at a level of public health concern (i.e., > 20%), probably because of the high proportion of children (i.e., 68%) reportedly receiving a bi-annual dose of vitamin A in the last 6 months, a finding consistent with recent national survey data [1]. The very high prevalence of vitamin D deficiency among our sample of toddlers is not unexpected, as comparable findings have been reported among children living in urban slum settings in New Delhi [61] where sunlight exposure is often limited [62] and air pollution is severe [63]. For example, in earlier studies of young children in Delhi, 73% at six months [64] and more than 80% of the same children followed up at 3–6 years were reported to be vitamin D deficient [13]. In addition, blood was collected at one point in March (spring) where vitamin D status may be lower than the summer months.

We observed a positive significant indirect association between vitamin D and hemoglobin that was largely explained by a strong soluble transferrin receptor. Co-existing deficiencies of vitamin D and iron were prevalent (n = 43/77, 55.8%) in our study. Possible mechanisms whereby vitamin D could influence iron metabolism and subsequently hemoglobin include stimulation of erythropoietic cells and inhibition of inflammatory cytokines and hepcidin production [65], albeit the directional relationship is unknown due to the observational nature of the study. In vitamin D deficiency, inflammatory cytokines stimulate an increase in circulating hepcidin levels which block the release of iron from body stores [66], so that a subsequent increase in soluble transferrin receptors would not be unexpected.

Lastly, approximately one third of the toddlers had evidence of folate or vitamin B12 deficiency, consistent with other studies in New Delhi children [9, 10, 12], but no evidence of macrocytic anemia based on an elevated mean cell volume. Hence, despite earlier reports of positive associations between concentrations of folate and vitamin B12 and hemoglobin among Indian children [12, 67], no association was observed here after adjusting for other micronutrients and non-nutritional determinants of anemia.

This is one of the first studies in young Indian children to examine seven micronutrient biomarkers and their interrelationships with hemoglobin and anemia, although our cross-sectional design precludes causal inferences being made. Moreover, because our results are based on a small convenience sample of children, they are not representative of toddlers living in urban slums of New Delhi. Given the small sample we have been careful to examine associations based not just on statistical significance but strength of association. We have also adjusted the micronutrient biomarkers influenced by inflammation by the new BRINDA regression modelling [14] and generated correct prevalence estimates for these micronutrients in a setting where the burden of subclinical inflammation is high. Nevertheless, genetic hemoglobin disorders, which are prevalent in India [68], have the potential to confound total body iron assessment by elevating sTfR in response to an increased rate of erythropoiesis [22]. As a result, they can lower hemoglobin concentrations and have been implicated in anemia [69]. Because we did not measure hemoglobinopathies, we were unable to assess the extent to which these conditions contribute to elevated sTfR.

In conclusion, our findings highlight that the majority of these Indian toddlers living in the urban slums of New Delhi were anemic, and iron deficiency was the largest contributor to nutritional anemia. Nonetheless, over half were at risk of three or more co-existing micronutrient deficiencies; and, while neither vitamin A, zinc, folate nor vitamin B12 were related to hemoglobin concentrations, such deficiencies have far reaching consequences on infant and child health. Clearly a coordinated multi-micronutrient program is urgently needed to combat the co-existing micronutrient deficiencies in these toddlers. Educating mothers on infant and young child feeding practices and use of micronutrient powders (MNP) for home fortification of foods is likely to have significant impact given the established benefits on hematological outcomes and iron status [70]. Moreover, delivery of MNP through the Integrated Child Development Services (ICDS) in India has shown to improve hemoglobin status among young children [71].
